# Synergizing Immunotherapy and Antibody–Drug Conjugates: New Horizons in Breast Cancer Therapy

**DOI:** 10.3390/pharmaceutics16091146

**Published:** 2024-08-29

**Authors:** Antonello Pinto, Chiara Guarini, Marianna Giampaglia, Valeria Sanna, Assunta Melaccio, Laura Lanotte, Anna Natalizia Santoro, Francesca Pini, Antonio Cusmai, Francesco Giuliani, Gennaro Gadaleta-Caldarola, Palma Fedele

**Affiliations:** 1 Oncology Unit, “Dario Camberlingo” Hospital, 72021 Francavilla Fontana, Italy; chiara.guarini@asl.brindisi.it (C.G.); annanatalizia.santoro@asl.brindisi.it (A.N.S.); francesca.pini@asl.brindisi.it (F.P.); 2 Oncology Unit, “San Carlo” Hospital, 85100 Potenza, Italy; mariannagiampaglia@yahoo.it; 3 Oncology Unit, “Ospedale Civile Santissima Annunziata” Hospital, 07100 Sassari, Italy; valeria.sanna@aouss.it; 4 Oncology Unit, “San Paolo” Hospital, 70123 Bari, Italy; assunta.melaccio@asl.bari.it (A.M.); francesco.giuliani@asl.bari.it (F.G.); 5 Oncology Unit, “Mons. Dimiccoli” Hospital, 70051 Barletta, Italy; laura.lanotte@aslbat.it (L.L.); gennaro.gadaleta@aslbat.it (G.G.-C.); 6 “Don Tonino Bello”, I.R.C.C.S. Istituto Tumori “Giovanni Paolo II”, 70124 Bari, Italy; antoniocusmai@hotmail.com

**Keywords:** immunotherapy, antibody–drug conjugates (ADCs), breast cancer, resistance mechanisms, synergistic effects

## Abstract

The advent of immunotherapy and antibody–drug conjugates (ADCs) have revolutionized breast cancer treatment, offering new hope to patients. However, challenges, such as resistance and limited efficacy in certain cases, remain. Recently, the combination of these therapies has emerged as a promising approach to address these challenges. ADCs play a crucial role by delivering cytotoxic agents directly to breast cancer cells, minimizing damage to healthy tissue and enhancing the tumor-killing effect. Concurrently, immunotherapies harness the body’s immune system to recognize and eliminate cancer cells. This integration offers potential to overcome resistance mechanisms and significantly improve therapeutic outcomes. This review explores the rationale behind combining immunotherapies with ADCs, recent advances in this field, and the potential implications for breast cancer treatment.

## 1. Introduction

**Figure 1:**
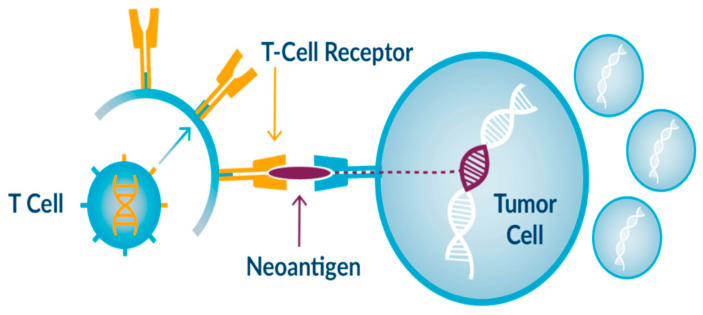
Example of immunotherapy drug action.

**Figure 2:**
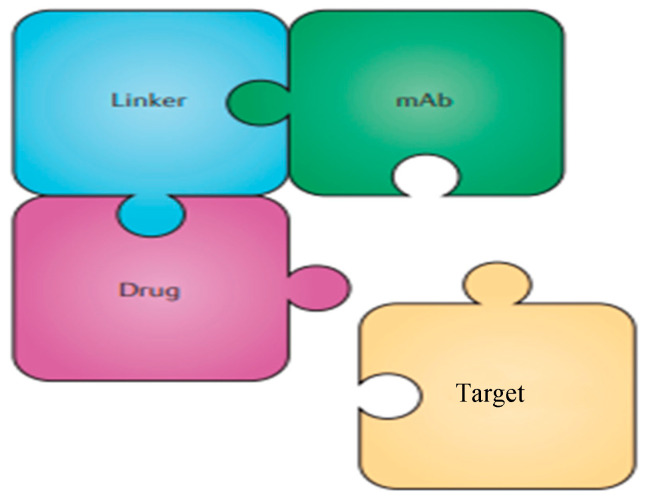
Simplified structure of an ADC directed against a given target.

In recent years, the combination of antibody–drug conjugates (ADCs) with immuno-oncology (IO) therapies has emerged as a promising strategy in the treatment landscape of breast cancer [1]. ADCs, designed to deliver cytotoxic agents selectively to tumor cells while sparing healthy tissues, complement the systemic approach of IO therapies, particularly immune checkpoint inhibitors (ICIs). Antibody–drug conjugates (ADCs) are typically composed of a monoclonal antibody (mAbs) covalently attached to a cytotoxic drug via a chemical linker. They combine both the advantages of highly specific targeting ability and highly potent killing effect to achieve accurate and efficient elimination of cancer cells, which has become one of the hotspots for the research and development of anticancer drugs. Since the first ADC, Mylotarg (gemtuzumab ozogamicin), was approved in 2000 by the US Food and Drug Administration (FDA), 14 ADCs have received market approval so far worldwide. Moreover, over 100 ADC candidates have been investigated in clinical stages at present. This type of new anticancer drug, known as “biological missiles”, is leading a new era of targeted cancer therapy. Herein, we conduct a review of the history and general mechanism of action of ADCs, and then briefly discuss the molecular aspects of key components of ADCs and the mechanisms by which these key factors influence the activities of ADCs [2]. The advent of immune checkpoint inhibition (ICI) using antibodies against PD1 and its ligand PD-L1 has prompted substantial efforts to develop complementary drugs. Although many of these are antibodies directed against additional checkpoint proteins, there is an increasing interest in small-molecule immuno-oncology drugs that address intracellular pathways, some of which have recently entered clinical trials. In parallel, small molecules that target pro-tumorigenic pathways in cancer cells and the tumor microenvironment have been found to have immunostimulatory effects that synergize with the action of ICI antibodies, leading to the approval of an increasing number of regimens that combine such drugs. Combinations with small molecules targeting cancer metabolism, cytokine/chemokine and innate immune pathways, and T cell checkpoints are now under investigation [3] (Figure 1 and Figure 2). This strategic combination aims to synergistically enhance therapeutic efficacy by leveraging ADCs’ ability to induce immunogenic cell death and promote T cell infiltration into tumors [4,5]. These mechanisms not only target tumor cells directly but also activate the host immune response against cancer, potentially overcoming resistance mechanisms observed with single-agent therapies. The evolving clinical landscape has seen a spectrum of trials exploring various ADC–ICI combinations across different breast cancer subtypes, reflecting the growing interest in optimizing treatment regimens [6]. Despite the advances, challenges remain in achieving durable responses and overcoming resistance, which this review aims to address by exploring the synergy between ADCs and ICIs. While outcomes from these trials vary, ongoing research continues to refine and expand our understanding of how these combined approaches can best serve patients, offering new avenues for personalized and effective breast cancer therapies.

This review synthesizes findings from clinical trials exploring various ADC–ICI regimens across different breast cancer subtypes.

## 2. The Rationale for Combining Antibody–Drug Conjugates (ADCs) and Immune Checkpoint Inhibitors (ICIs) in Breast Cancer

Breast cancer remains a significant clinical challenge, necessitating the development of newer and more effective systemic anticancer agents. Antibody–drug conjugates (ADCs) have demonstrated significant efficacy, with the US FDA and EMA approving trastuzumab emtansine, trastuzumab deruxtecan, and sacituzumab govitecan for different breast cancer settings. These ADCs target specific antigens on breast cancer cells, delivering cytotoxic drugs directly to the tumor, thereby minimizing systemic toxicity [7].

Immune checkpoint inhibitors (ICIs), such as atezolizumab and pembrolizumab, have shown encouraging activity and are approved by the US FDA and EMA for patients with PD-L1-positive breast cancer. ICIs work by blocking inhibitory pathways in T cells, thereby enhancing the immune system’s ability to attack cancer cells [8]. However, the efficacy of ICIs in breast cancer has been limited to a subset of patients, indicating a need for strategies to enhance their effectiveness.

The combination of ADCs and ICIs is of significant scientific and clinical interest due to the complementary mechanisms of these therapies, which together can potentially provide a more robust and durable anti-tumor response. ADCs are designed to deliver cytotoxic agents directly to cancer cells by targeting specific antigens, leading to the selective killing of tumor cells and minimizing damage to normal tissues. This targeted approach not only improves the efficacy of the cytotoxic agent but also enhances the immunogenicity of the tumor [9].

One of the key mechanisms by which ADCs can enhance the effectiveness of ICIs is through the induction of immunogenic cell death (ICD). When ADCs induce ICD, they cause the release of damage-associated molecular patterns (DAMPs) and tumor-associated antigens (TAAs). This process enhances the presentation of these antigens to dendritic cells and other antigen-presenting cells (APCs), thereby stimulating a stronger and more specific immune response. The increased availability of TAAs can improve the activation and proliferation of T cells, particularly when combined with ICIs, which block inhibitory signals and unleash the immune system’s full potential [10].

This synergy is particularly important in overcoming resistance mechanisms that limit the efficacy of monotherapies. For instance, tumors often develop mechanisms to evade immune detection, such as downregulating antigen presentation or creating an immunosuppressive microenvironment. ADCs can disrupt these evasion tactics by increasing antigen release and altering the tumor microenvironment. Preclinical studies have shown that ADC-induced cell death can lead to the infiltration of immune cells, such as cytotoxic T lymphocytes (CTLs) and natural killer (NK) cells, into the tumor. This infiltration is critical for an effective immune response and can be further enhanced by ICIs, which help sustain and amplify the activity of these immune cells [11].

Moreover, the cytotoxic effects of ADCs may lead to the release of neoantigens, which are novel antigens resulting from tumor-specific mutations. These neoantigens are highly immunogenic because they are recognized as foreign by the immune system. The presence of ICIs can enhance the recognition and destruction of neoantigen-bearing tumor cells by preventing the engagement of inhibitory receptors, such as PD-1, on T cells. This results in a more vigorous and sustained immune attack on the tumor [12].

In addition to direct tumor cell killing, ADCs can modulate the tumor microenvironment to make it more conducive to immune cell infiltration and activity. For example, the destruction of tumor cells by ADCs can reduce the immunosuppressive cell populations, such as regulatory T cells (Tregs) and myeloid-derived suppressor cells (MDSCs), within the tumor microenvironment. This reduction in immunosuppression can further enhance the efficacy of ICIs by allowing a more effective immune response [13].

Clinical trials exploring the combination of ADCs and ICIs are underway and have shown promising early results. These trials indicate that the combination can lead to improved response rates and longer progression-free survival in patients with breast cancer. The rationale for this combination lies in the ability of ADCs to prime the immune system and enhance antigen presentation, while ICIs remove the brakes on the immune system, allowing for a more comprehensive and effective anti-tumor response [14].

## 3. Efficacy Data of Immune Checkpoint Inhibitors and ADCs in Early Breast Cancer

Recent advancements in the field of oncology have introduced promising treatments, notably ICIs and ADCs. These therapies have shown significant potential in improving treatment outcomes for patients with early breast cancer.

Herein, we report key studies conducted on immunotherapy and highlight the most interesting results regarding drug response and survival benefits.

### 3.1. Immunotherapy in Early Breast Cancer

Triple-negative breast cancer (TNBC), characterized by the absence of estrogen and progesterone receptors and lack of HER2 protein expression, represents one of the most aggressive and challenging subtypes of breast cancer. Despite the aggressive nature of TNBC, recent advances in immunotherapy and ADCs offer hope for improved outcomes.

The KEYNOTE-522 [15] trial investigated the efficacy of pembrolizumab, an anti-PD-1 antibody, in combination with chemotherapy as a neoadjuvant treatment for early-stage TNBC. The study included patients with stage II or III TNBC who received pembrolizumab or placebo alongside chemotherapy before surgery, followed by adjuvant pembrolizumab or placebo. The results were groundbreaking, demonstrating a significant improvement in pathological complete response (pCR) rates. The trial reported that patients receiving the combination therapy had a pCR rate of 64.8%, compared to 51.2% in the chemotherapy-alone group. Additionally, the event-free survival (EFS) rate at 18 months was notably higher in the pembrolizumab group (85.3%) versus the control group (77.9%). The increased pCR rates suggest that adding immunotherapy to standard chemotherapy can enhance the eradication of cancer cells before surgery, potentially improving long-term outcomes.

The IMpassion031 [16] trial assessed atezolizumab, an anti-PD-L1 antibody, combined with chemotherapy in early TNBC. The trial revealed a significant improvement in pCR rates, with 58% in the atezolizumab group versus 41% in the placebo group. This study underscored the potential of combining immunotherapy with standard treatments to enhance therapeutic efficacy. Similar to KEYNOTE-522, this study found that the addition of atezolizumab significantly increased pCR rates compared to chemotherapy alone. 

Similarly, the NEOTRIP [17] trial evaluated the combination of atezolizumab, a PD-L1 inhibitor, with chemotherapy for neoadjuvant treatment of TNBC. Although the primary endpoint of increased pCR was not met, secondary analyses suggested potential benefits in specific subgroups of patients.

The GEPARNEUVO [18] trial evaluated the use of durvalumab, another PD-L1 inhibitor, in combination with chemotherapy in the neoadjuvant setting. The study focused on assessing whether adding durvalumab could increase pCR rates in patients with TNBC. The findings revealed that patients receiving durvalumab plus chemotherapy had higher pCR rates compared to those receiving chemotherapy alone. 

These studies underscored the complexity of TNBC and the need for further research to identify which patients might benefit most from the addition of immunotherapy. They also highlighted the importance of biomarker-driven approaches to optimize treatment strategies.

As regards adjuvant setting, the ALEXANDRA (IMpassion030) [18] trial investigated the addition of atezolizumab to standard chemotherapy in early-stage triple-negative breast cancer (TNBC) patients. Despite enrolling 2199 patients, the trial found no improvement in invasive disease-free survival (iDFS) with the addition of atezolizumab compared to chemotherapy alone. The hazard ratios for iDFS and overall survival showed no significant benefit in the intention-to-treat population or subgroups analyzed. The safety profile of atezolizumab remained consistent, with higher rates of grade 3 or greater adverse events in the atezolizumab group. Ultimately, the study concluded that atezolizumab did not enhance treatment outcomes in this setting. 

The A-Brave trial [19] evaluated avelumab as an adjuvant treatment for high-risk early-stage TNBC patients. This phase III study compared a year of avelumab treatment to observation after standard therapy. Despite no significant improvement in disease-free survival (DFS) with avelumab, overall survival (OS) was significantly better in the avelumab group

As for HER2-positive breast cancer, characterized by the overexpression of the HER2 protein, recent advances in immunotherapy are showing significant promise in both neoadjuvant and adjuvant settings.

The APTneo trial [20] tested the addition of atezolizumab to neoadjuvant HER2-targeted therapy with trastuzumab, pertuzumab, and chemotherapy in HER2+ breast cancer. Involving 661 patients, the study compared different treatment combinations to assess their impact on pathologic complete response (pCR). While no significant pCR difference was found between the main arms, Arm B1 (with anthracyclines) showed a 9.9% higher pCR than Arm A. Atezolizumab was well tolerated, and the enhanced effect in Arm B1 might be due to anthracyclines or immune modulation. Patients will continue to be monitored for long-term event-free and overall survival outcomes.

The phase III IMpassion050 [21] trial evaluated the addition of atezolizumab to standard treatment in high-risk HER2-positive early breast cancer. Patients were randomly assigned to receive either atezolizumab or a placebo alongside chemotherapy and HER2-targeted therapy. The study found no significant increase in pathologic complete response (pCR) rates with atezolizumab in either the overall or PD-L1-positive populations compared to placebo. Grades 3–4 and serious adverse events were more common in the atezolizumab group, including some fatal events. The standard of care remains pertuzumab-trastuzumab and chemotherapy, with future follow-up needed for long-term effects of atezolizumab.

Hormone-receptor-positive (HR-positive) breast cancer, which expresses estrogen and/or progesterone receptors, is the most common subtype of breast cancer. While endocrine therapy has significantly improved outcomes, there remains a need for additional treatments, particularly for patients with high-risk early-stage disease.

The KEYNOTE-756 trial is a crucial study investigating the use of pembrolizumab, an anti-PD-1 antibody, in combination with neoadjuvant endocrine therapy for patients with high-risk, HR-positive, HER2-negative early breast cancer. The trial aims to assess whether the addition of pembrolizumab can improve pCR rates and EFS. Preliminary results suggest that pembrolizumab may enhance the efficacy of neoadjuvant endocrine therapy, potentially leading to higher pCR rates. This combination could represent a significant step forward in the treatment of high-risk HR-positive breast cancer, offering an effective approach to reduce the likelihood of recurrence and improve long-term outcomes [22,23].

The CHECKMATE-7FL trial evaluates the efficacy of nivolumab, an anti-PD-1 antibody, in combination with neoadjuvant endocrine therapy for HR-positive, HER2-negative early breast cancer. This study focuses on improving pCR rates and DFS by harnessing the body’s immune system to target cancer cells more effectively [24]. Early data from CHECKMATE-7FL indicate promising improvements in pCR rates for patients receiving nivolumab in addition to standard neoadjuvant endocrine therapy. These findings suggest that immunotherapy can play a significant role in enhancing the effectiveness of existing treatments for HR-positive breast cancer.

### 3.2. Antibody–Drug Conjugates in Early Breast Cancer

Antibody–drug conjugates (ADCs) represent a novel class of targeted therapies that combine monoclonal antibodies with cytotoxic drugs. These conjugates specifically target cancer cells, delivering cytotoxic agents directly to the tumor site, thereby minimizing systemic toxicity.

The KATHERINE trial is a landmark study for HER2-positive breast cancer, which can also be ER-positive, in the adjuvant setting for patients with residual invasive disease post-neoadjuvant therapy. This phase 3 trial demonstrated a significant improvement in invasive disease-free survival (iDFS) with T-DM1 compared to trastuzumab. The 3-year iDFS rate was 88.3% for T-DM1 versus 77.0% for trastuzumab, indicating a substantial benefit in reducing the recurrence risk [25].

Sacituzumab govitecan targets Trop-2 and has shown promise in early TNBC. The NeoSTAR study evaluated this ADC in the neoadjuvant setting, reporting an impressive pathological complete response (pCR) rate of 45% in patients with TNBC. The study is ongoing, but these early results suggest that sacituzumab govitecan could significantly impact treatment outcomes for early TNBC [26].

Datopotamab deruxtecan, another Trop-2-targeting ADC, is currently being evaluated in the neoadjuvant setting for TNBC. The TROPION-Breast02 trial is currently investigating datopotamab deruxtecan in the neoadjuvant and adjuvant settings for HR+ breast cancer. Early results from the neoadjuvant cohort indicate a notable ORR of 44%, with ongoing follow-up to assess DFS and OS benefits. The trial aims to provide comprehensive data on the efficacy and safety of datopotamab deruxtecan in HR+ breast cancer [27].

The TROPION-Breast03 study is a phase III trial evaluating the efficacy of datopotamab deruxtecan (Dato-DXd), alone or with durvalumab, against standard therapies in early-stage triple-negative breast cancer (TNBC) patients with residual invasive disease after surgery. Patients are randomized to receive either Dato-DXd with durvalumab, Dato-DXd alone, or a treatment chosen by the investigators, including capecitabine and pembrolizumab. The primary endpoint is invasive disease-free survival (iDFS), with secondary endpoints assessing safety, distant disease-free survival, and overall survival. This trial aims to address the unmet need for improved adjuvant treatments in TNBC [28].

The IMMU-132-01 trial investigated sacituzumab govitecan in a subset of patients with HR+ early breast cancer in the neoadjuvant setting. This study demonstrated a promising overall response rate (ORR) of 33% in HR+ patients. Preliminary data suggest improvements in DFS, although long-term OS data are still awaited [29].

## 4. Efficacy Data of Immune Checkpoint Inhibitors and ADCs in Metastatic Breast Cancer

### 4.1. Efficacy Data of Immune Checkpoint Inhibitors in Metastatic Breast Cancer

TNBC are associated with poor prognosis and, until a few years ago, the only possible treatment in the metastatic setting was chemotherapy. TNBC is the breast cancer subtype with the highest rates of tumor-infiltrating lymphocytes (TILs), programmed death ligand 1 (PD-L1) expression, and tumor mutational burden, making it a prime candidate for immunoterapia [30,31]. Several studies have evaluated the efficacy of different immunotherapy agents combined with various chemotherapy drugs, with results that are not always concordant.

Immunotherapy with PD-(L)1-blocking agents combined with ChT improves prognosis in mTNBC, but responses are limited to a proportion of patients, and most will experience disease progression. The adenosine pathway has been demonstrated to limit anti-tumor activity in TNBC, making CD73, the adenosine-generating enzyme, an attractive target to enhance the efficacy of immunotherapy in this disease [32].

The first drug to be approved in the first-line treatment of patients with metastatic, unresectable, or locally advanced PD-L1-positive TNBC was atezolizumab. In 2019, in light of the results of the study Impassion130 [33], atezolizumab in combination with nabpaclitaxel obtained accelerated approval from the FDA and, subsequently, from the EMA. The phase III study enrolled 902 patients and randomized them to receive the combination of atezolizumab with nabpaclitaxel or a placebo. The study demonstrated a statistically significant improvement in PFS in the intention-to-treat (ITT) population (7.2 months vs. 5.5 months, *p* = 0.002) and in the PD-L1-positive subgroup (7.5 months vs. 5 months, *p* < 0.001). As regards overall survival, having not reached statistical significance in the ITT population, it was not evaluated in the PD-L1 subgroup; although in an exploratory analysis, there appeared to be a clinical benefit (mOS 25.4 months vs. 17.9 months).

A subsequent trial, Impassion131, evaluated the association of atezolizumab with weekly paclitaxel in similar population (651 patients), showing no improvement in PFS and OS [34].

These discordant results could be associated with several factors: steroid premedication, patient heterogeneity, or previous administration of taxanes in an early setting.

Another drug that received accelerated approval from the FDA in metastatic, unresectable, or locally advanced PD-L1-positive TNBC was pembrolizumab, after the results of the KEYNOTE-355 trial [35].

This study tested the addition of pembrolizumab plus the investigator’s choice of chemotherapy (nab-paclitaxel, paclitaxel, or gemcitabine-carboplatin) or placebo in 847 patients, demonstrating a significant benefit in the CPS ≥ 10 subgroup for PFS and OS (mPFS = 9.7 months versus 5.6 months, *p* = 0.0012; mOS = 23 months versus 16.1 months, *p* = 0.0185). The three trials (Impassion130, Impassion131, and KEYNOTE-355) previously cited excluded patients with early relapsing TNBC, who were instead enrolled in the IMpassion132 trial [36]. In this phase III study, the patients were randomized to receive atezolizumab in combination with various chemotherapy agents (gemcitabine and capecitabine) or a placebo. Unfortunately, the combination with atezolizumab has not been shown to improve OS (primary endpoint) in the PDL-1-positive subgroup (mOS = 12.1 months versus 11.2 months in the placebo arm).

Several studies observed high levels of tumor-infiltrating lymphocytes (TILs) and of PD-L1 [37] in HER2-positive breast cancers, demonstrating a correlation between high levels of TILs with better prognosis, higher rates of pathological complete response (pCR), disease-free survival (DFS), and OS [38]. Differently, PD-L1 expression seems to associate with resistance to anti-HER2 agents, which can be overcome with checkpoint inhibition combinations [39].

The phase 1b/2 study PANACEA enrolled patients with HER2-positive advanced breast cancer progressing after trastuzumab-based therapy in two subgroups (PD-L1-positive and PD-L1-negative) to receive pembrolizumab plus trastuzumab.

The trial demonstrated that the association was safe and effective in the PD-L1-positive subgroup, obtaining a 15% objective response rate (ORR) [40].

Another trial, KATE2, a phase 2 trial, investigated the combination of atezolizumab and T-DM1 in patients with HER2-positive advanced breast cancer previously treated with trastuzumab.

The study demonstrated that the association did not improve PFS but increased side effects. Although, a positive trend in PFS and OS was observed in PD-L1-positive patients, suggesting a role of T-DM1 in eliciting anti-tumor immunity and acting as a primer to sensitize tumors to immune checkpoint inhibitors [41].

This hypothesis led to the development of new studies currently ongoing, such as the phase 3 KATE3 (NCT04740918) trial [42], which is investigating the same combination in the same setting but only in PD-L1-positive patients, with PFS and OS as co-primary endpoints.

Other ongoing studies, such as NCT03125928 (phase IIA) and NRG-BR004 (phase III trial, randomized and double-blind), will evaluate the safety and efficacy of atezolizumab in combination with paclitaxel, trastuzumab, and pertuzumab in patients with locally advanced, unresectable, or metastatic HER2-positive breast cancer in the first-line setting.

Hormone-receptor-positive (HR+) breast cancer subtypes have low tumor mutational burden (TMB), low PD-L1 expression, and low numbers of TILs [43,44]. However, several studies in recent years have evaluated the effectiveness of immunotherapy in this subgroup, demonstrating that cytotoxic drugs enhance tumor immunity. It appears that after exposure to chemotherapy, the release of tumor cell neoantigens may activate an anti-tumor immune response by inducing the infiltration and activation of CD8+ T cells [45]. The first of these studies is a phase II study, which evaluated the combination of eribulin mesylate (E) with or without pembrolizumab (P). The study enrolled 88 patients with multi-treated HR+/HER2− metastatic breast cancer (at least two previous lines of endocrine therapy and up to two lines of chemotherapy).

Unfortunately, the combination did not bring any benefit in median PFS (4.1 vs. 4.2 months, *p* = 0.38) [46].

Preclinical evidence has suggested that PARP inhibitors may have a role in upregulating the host anti-tumor immune response by increasing PD-L1 expression. The phase II MEDIOLA study evaluated this hypothesis by testing the efficacy of the combination of durvalumab (anti-PD-L1) with olaparib in 34 patients with metastatic breast cancer and BRCA 1/2 germline mutation (13 HR+ and 21 triple-negative disease) [47]. The study had two primary endpoints: the disease control rate (12-week DCR) and safety. Secondary endpoints were DCR at 28 weeks, ORR, PFS, and OS. The combination proved to be effective, achieving a DCR of 85% with a median PFS of 8.2 months, with better results in patients undergoing fewer lines of treatment (11.7 months in patients with 0–1 prior lines of chemotherapy vs. 6.5 months in 2 prior lines).

The OS benefit was similar between the two subtypes (HR+ = 22.4 months and TNBC = 20.5 months).

Preclinical studies have also highlighted an upregulation of immune-related genes in breast cancers with the invasive lobular histotype (ILBC) [48]. For this reason, the phase II GELATO study enrolled 26 patients with metastatic breast cancer with this histotype (ILBC) to evaluate the activity of carboplatin (weekly) in immune induction for 12 weeks, plus atezolizumab from the third week until progression, resulting in a clinical benefit rate of 26% (6 patients; from these 6 patients, 4 had triple-negative BC) [49].

The other study, phase II SAFIR02-BREAST IMMUNO, investigated the role of immunotherapy in maintenance therapy compared to chemotherapy alone. Here, 199 patients with metastatic HER2-BC who had not progressed after 6–8 cycles of chemotherapy (first or second line) were randomized to receive durvalumab or chemotherapy treatment.

The trial failed to demonstrate the primary endpoints of PFS (mPFS: 2.7 vs. 4.6 months) and OS benefits in the ITT population [50].

Considering the results of these studies, there are currently no FDA-approved immunotherapy agents for the treatment of HR+ breast cancer.

### 4.2. Efficacy Data of Antibody Drug Conjugates in Metastatic Breast Cancer

Several studies have investigated the efficacy and safety of ADCs in various subgroups of breast cancer. Trastuzumab deruxtecan (DS-8201) stands out among antibody–drug conjugates (ADCs) due to its composition, which includes a humanized monoclonal antibody that targets HER2 with the same amino acid sequence as trastuzumab. This ADC also features a tetrapeptide-based linker that can be cleaved and a highly potent topoisomerase I inhibitor as its cytotoxic agent [51].

The DESTINY-Breast04 study evaluated trastuzumab deruxtecan in patients with predominantly hormone-receptor-positive, HER2-low metastatic breast cancer, who had received one or two prior lines of chemotherapy. These patients, with HER2 1+ or 2+ status, were randomized (2:1) to receive either chemotherapy or trastuzumab deruxtecan, which is approved for HER2+ metastatic breast cancer. The results showed significant benefits in the hormone-receptor-positive cohort (median PFS: 10.1 months vs. 5.4 months; OS: 23.9 months vs. 17.5 months). Among all patients, median PFS was 9.9 months in the ADC group versus 5.1 months in the chemotherapy group, and overall survival was 23.4 months versus 16.8 months, respectively [52].

The DESTINY-Breast04 study established that trastuzumab deruxtecan is best utilized following first-line chemotherapy for both HR-positive and triple-negative breast cancer. However, it is anticipated that 10–15% of patients receiving T-DXd may experience interstitial lung disease, with a fatality rate of 1–2% among these cases [53].

The safety and efficacy of another ADC, sacizumab govitecan, was tested in patients with endocrine-resistant and chemotherapy-resistant HR+ and HER2– metastatic breast cancer in the TROPICS 02 pivotal trial [54,55,56].

Researchers found that participants treated with sacituzumab govitecan had a higher overall survival rate compared to those who received chemotherapy. In these patients, the median progression-free survival was 5.5 months, with a 34% reduction in the risk of disease. Additionally, a greater percentage of tumors shrank by at least 30% or disappeared entirely in the sacituzumab govitecan group. This treatment was effective regardless of Trop-2 levels and HER2 status in the cancer cells.

Trophoblast cell surface antigen 2 (TROP2) is a transmembrane protein with low expression in normal tissues but high levels in HR+/HER2– and triple-negative breast cancer (TNBC), often indicating a poor prognosis. This makes TROP2 an attractive target for therapeutic interventions in these breast cancer subtypes. Specifically, TROP2 is the target of the antibody–drug conjugate (ADC) datopotamab deruxtecan (Dato-DXd), which consists of a humanized anti-TROP2 monoclonal antibody linked to a potent topoisomerase I inhibitor (a hexatecan derivative) via a cleavable linker that is stable in plasma but selectively broken down in tumors. This design mitigates systemic side effects and off-target toxicity. When Dato-DXd binds to TROP2-expressing tumor cells, it induces their death and triggers apoptosis in adjacent cells within the tumor environment [57,58,59,60,61,62,63].

The TROPION-Breast01 trial (NCT05104866) is a phase III study evaluating the safety and efficacy of Dato-DXd compared to standard single-agent chemotherapy chosen by the investigator. This trial focuses on patients with inoperable or metastatic HR+/HER2− breast cancer who have previously received one or two lines of systemic chemotherapy [64].

The first results of this study demonstrated that the vast majority of patients receiving Dato-DXd had significantly improved PFS vs. ICC (HR 0.63 (95% CI 0.52–0.76); *p* < 0.0001). OS data were not mature, and a trend for improvement favoring Dato-DXd was observed. Patients receiving Dato-DXd had lower rates of grade ≥3 TRAEs and dose reductions vs. ICC [65].

These results demonstrate that in this patient setting, ADCs represent a concrete new treatment option over traditional chemotherapy.

HER2+ patients have also been heavily investigated concerning the effectiveness of ADCs for some time. In the single-arm DESTINY-Breast01 phase II clinical trial, T-DXd showed clinical activity in the third-line setting for patients with HER2-positive, heavily pretreated metastatic breast cancer. These results led to the accelerated approval of T-DXd in 2019 as a third-line therapy for patients with metastatic or unresectable breast cancer who have received two or more prior HER2-targeted therapies [66]. The DESTINY-Breast02 trial is the first randomized trial to assess the efficacy of one antibody–drug conjugate, T-DXd, in patients whose cancer has already progressed on another antibody–drug conjugate, T-DM1 treatment. In this study, 608 patients whose metastatic breast cancers had progressed on or after T-DM1 treatment were enrolled and randomized 2:1 to receive either T-DXd or the physician’s choice (a combination of capecitabine with either trastuzumab or lapatinib). In patients receiving T-DXd treatment, 69.7% showed an objective response, significantly higher than the 29.2% observed in those receiving treatment chosen by their physician. Additionally, the likelihood of disease progression was reduced by 64% in the T-DXd group compared to the physician’s choice group. The median progression-free survival was 17.8 months for the T-DXd group, versus 6.9 months for those receiving the physician’s chosen treatment. Furthermore, the overall survival rate was notably higher in the T-DXd group, with patients living an average of 39.2 months, compared to 26.5 months for those in the physician’s choice group.

Adverse events in patients who received T-DXd were consistent with prior studies [67]. A limitation of this study was that the control arm was limited to therapies based on capecitabine, precluding direct comparison of T-DXd to treatment regimens containing other chemotherapeutic agents. An additional limitation is that patients with progressive metastases to the central nervous system were not eligible for the trial. Subsequently, the aim of the DESTINY-Breast03 study was to compare the efficacy and safety between trastuzumab deruxtecan and trastuzumab emtansine [68]. The study reported an impressive median progression-free survival (PFS) of 28.8 months, with a median treatment duration of 18.2 months in patients treated with trastuzumab deruxtecan. Notably, 21.4% of treatment discontinuations were due to adverse events, with 12.7% specifically attributed to various forms of pulmonary toxicity, such as interstitial lung disease or pneumonitis [69]. The results were also excellent in overall survival (hazard ratio 0.64, 95% CI 0.47–0.87; *p* = 0.0037; overall survival at 24 months: 77.4% vs. 69.9%). The authors concluded that the overall survival data reaffirmed trastuzumab deruxtecan as the standard of care in the second-line setting. ADCs have, therefore, proven to be particularly useful so far as post-first-line therapy in HER2-positive patients, although there are several ongoing studies aimed at testing the drug in the first line and early stages (TRANSCENDER, NCT05744375, and SHAMROCK studies) [70,71].

Patients with triple-negative metastatic breast cancer typically face poor survival rates. Although immunotherapy is emerging as a promising first-line treatment, single-agent chemotherapy remains the standard for those who have already undergone treatment. However, these standard treatments often result in low response rates and brief periods of progression-free survival [72,73,74,75,76]. The Phase III ASCENT trial evaluated the efficacy of the antibody–drug conjugate (ADC) sacituzumab govitecan against the physician’s choice of single-agent chemotherapy (including eribulin, vinorelbine, capecitabine, or gemcitabine) in patients with relapsed or refractory metastatic triple-negative breast cancer. The trial revealed that sacituzumab govitecan, which targets Trop-2 and delivers the topoisomerase inhibitor SN-38 [77], significantly enhanced survival outcomes compared to traditional chemotherapy. Specifically, the trial reported a median progression-free survival (PFS) of 5.6 months versus 1.7 months, a median overall survival (OS) of 12.1 months versus 6.7 months, an overall response rate (ORR) of 35% versus 5%, a clinical benefit rate (CBR) of 45% versus 9%, and a median duration of response (DOR) of 6.3 months versus 3.6 months. These results led to the US Food and Drug Administration (FDA) approving sacituzumab govitecan in 2021 for adults with locally advanced, unresectable or metastatic triple-negative breast cancer who have received at least two prior systemic therapies, including one for metastatic disease [78,79,80]. Further studies, such as NeoSTAR (NCT04230109) and SASCIA (NCT04595565), are ongoing to explore the potential of sacituzumab govitecan as an earlier treatment option for breast cancer [81,82]

In the DESTINY-Breast04 study, T-DXd improved progression-free and overall survival in patients with metastatic breast cancer with HER2-low tumors (defined as HER2 1+ or 2+ and not amplified by in situ hybridization (ISH)), including the triple-negative subgroup (58 patients). This led to the approval of T-DXd for HER2-low metastatic triple-negative breast cancer, excluding HER2 0, highlighting the clinical importance of detecting HER2-low status.

While HER2-low status is detected in about 30%–50% of triple-negative breast cancer patients, various studies have shown that HER2 expression is heterogeneous and can change over time. However, the benefit of repeated biopsies in detecting new HER2-low status for patients with triple-negative disease is currently unknown [83].

In conclusion, thanks to these studies, we currently have two potentially effective ADCs in metastatic TN breast cancer. Researchers will probably have to better identify the patient settings in which these drugs should be allocated, considering the “variable” biological characteristics of the tumors and their positions in the therapeutic algorithm.

## 5. Clinical Efficacy of Immunotherapy and Antibody–Drug Conjugates in Breast Cancer: Evidence from Trials

Clinical studies aimed at investigating the combinations of ADCs and ICIs are underway; among these, the BEGONIA study [84] is evaluating the combination of chemotherapy with immune checkpoint inhibitors and novel therapies for metastatic triple-negative breast cancer (TNBC). Initial results from Part 1 of the study show that Durvalumab (D) plus Paclitaxel (P) had a 57% objective response rate (ORR) with a median progression-free survival (PFS) of 7.3 months. In contrast, Durvalumab plus T-DXd, an antibody–drug conjugate targeting HER2, showed a 100% ORR in a small group of patients with HER2-low tumors, with promising early safety results. Arm 1 (D + P) had manageable toxicity, while Arm 6 (D + T-DXd) showed no dose-limiting toxicities. The study is ongoing, with further evaluations and updates expected for both treatments.

Preliminary data from the DESTINY-Breast07 study were recently presented at ASCO 2024 [85]. The study evaluated T-DXd alone and in combination with pertuzumab (P) as first-line treatments for HER2+ mBC. Interim results show a high objective response rate (ORR) of 77.3% for T-DXd and 82.0% for T-DXd + P, with 12-month PFS rates of 77.3% and 89.4%, respectively. Common adverse events include nausea and diarrhea, with no grade 4 events reported. Both treatments demonstrated promising efficacy and manageable safety profiles, and the study is ongoing.

The I-SPY2.2 trial is a phase 2 study designed to evaluate novel neoadjuvant breast cancer regimens, focusing on achieving pathological complete response (pCR) with targeted agents, followed by standard treatments, if needed. It uses a predictive subtype classification (RPS) that includes immune, DNA repair deficiency (DRD), and luminal signatures to tailor treatments. Patients in RPS S1–S4 were assigned to receive Dato + Durva in the first treatment block, followed by either taxane-based regimens or additional therapies based on their response. Initial results showed that Dato + Durva achieved a 72% pCR rate in the RPS S3 subtype, suggesting its potential effectiveness. Further investigation in a larger trial is recommended to confirm these findings [86].

The phase 2 SACI-IO study evaluated the efficacy of SG combined with pembrolizumab (a PD-1 inhibitor) versus SG alone in HR+/HER2− metastatic breast cancer. The study found that the combination therapy had a non-significant trend toward improved progression-free survival (PFS) compared to SG alone, with median PFS of 8.4 months versus 6.2 months. The objective response rate (ORR) was similar between the two arms. While preliminary results suggest potential benefits, final PFS and overall survival data are still pending further follow-up [87].

Currently, as indicated in Table 1, there are several ongoing trials assessing the effectiveness and safety of ADCs combined with ICIs (Table 1).

## 6. Conclusions

The integration of immunotherapy and antibody–drug conjugates (ADCs) in breast cancer treatment shows promising potential, offering enhanced efficacy through synergistic mechanisms. Clinical trials demonstrate improved outcomes, suggesting these combinatorial approaches may overcome resistance and increase survival rates. Future research should focus on optimizing treatment protocols to maximize patient benefits. ADCs, therefore, represent a revolutionary approach in cancer therapy, ensuring a method of administration that guarantees less toxicity at the level of healthy tissues. Following the approval of the first ADCs against breast cancer, development at the pharmaceutical and biotechnological levels has seen the birth of new ADCs of subsequent generations, much more specific and complete from a structural and functional point of view. However, several clinical issues remain to be addressed, one of which is overcoming the resistance mechanisms in patients treated with ADCs. In this sense, the combined approach between ADCs and ICIs has taken hold and continues to be an effective strategy, identifying specific molecular markers unique to each individual patient, thus reducing the possible combined side effects and circumventing the mechanisms of resistance to therapy. The future from this point of view is full of new methodologies, with studies focused on restoring favorable clinical conditions for patients and the development of drugs with even more specific molecular targets.

## Figures and Tables

**Table 1:** Ongoing trials assessing the effectiveness and safety of ADCs combined with ICIs.

ADC	ADC Target	Trials/Phase	Combination Therapy	Patient Population	Key Objective	NCT No.
Trastuzumab Emtansine (T-DM1)	HER2	KATE2 II	T-DM1+ atezolizumab	2L HER2+ MBC	PFS	NCT02924883 [88]
Trastuzumab deruxtecan (T-DXd)	HER2	I	Nivolumab	Advanced breast or urothelial Ca	DLT, ORR	NCT03523572 [89]
		IB	Pembrolizumab	Advanced breast cancer or NSCLC	DLT, ORR	NCT04042701 [90]
		DESTINY Breast07 I/II	Durvalumab or pertuzumab, or paclitaxel +/− durvalumab or tucatinib	HER2+ MBC	AEs	NCT04538742 [91]
Disitamab vedotin (RC48-ADC)	HER2		Penpulimab (AK105)	Neoadjuvant HER2-low BC	pCR	NCT05726175 [92]
ARX-788	HER2	ISPY-2.2 II	Cemiplimab	Neoadjuvant stage I–III HER2+ BC	pCR	NCT01042379 [93]
Sacituzumab Govitecan	TROP2	ASCENT 05 III	Pembrolizumab	Post-neoadjuvant stage I–III TNBC with residual	iDFS	NCT05633654 [94]
		ASCENT 04 III	Pembrolizumab	First-line PD-L1+ metastatic TNBC	PFS	NCT05382286 [95]
Dato-Dxd	TROP-2	TROPION-Breast03 III	Durvalumab	Post-neoadjuvant stage I–III TNBC with residual disease	iDFS	NCT05629585 [96]
		ISPY-2.2 II	Durvalumab	Neoadjuvant stage I–III TNBC	pCR	NCT01042379 [97]
Ladiratuzumab vedotin (SGN- LIV1A)	LIV-1	MORPHEUS-panBC II	Atezolizumab	Metastatic or locally advanced breast cancer	ORR	NCT03424005 [98]
		SGNLVA 002 or KEYNOTE-721 I/II	Pembrolizumab	Advanced TNBC	ORR	NCT03310957 [99]

PFS: progression-free survival; iDFS: invasive disease-free survival; AEs: adverse events; pCR: pathologic complete response; PR2D: recommended phase II dose; DLT: dose-limiting toxicities; ORR: overall response rate.
